# A huge absence of skin on the trunk: aplasia cutis congenita

**DOI:** 10.11604/pamj.2018.31.234.17744

**Published:** 2018-12-19

**Authors:** Pelin Dogan, Ipek Güney Varal

**Affiliations:** 1 Department of Pediatrics, Division of Neonatology, University of Health Sciences, Bursa Yüksek Ihtisas Teaching Hospital, Bursa, Turkey

**Keywords:** Aplasia cutis congenita, congenital anomalies, genetic

## Image in medicine

A 2590 g female infant was born at 39 weeks' gestation to a 34-year-old gravida 4 para 4 mother via cesarean section. Upon delivery she was noted to have an absence of skin on the huge part of the trunk. Lesion was unilateral, gelatinous and covered by a thin membrane. The pregnancy history was unremarkable, the mother denied having taken any drugs and there was no family history of congenital anomalies or consanguinity. The physical examination of the infant was otherwise unremarkable. Conservative treatment was recommended by dermatologist with gentle cleansing and application of local antibiotics. Ultrasonographic evaluation of abdomen and kranium, echocardiographic evaluation and detailed genetic assessment were normal. The patient was discharged home on full oral feedings on day 6 and recommended follow up at the outpatient dermatology clinic. Definition of aplasia cutis congenita (ACC) is, complete or partial absence or scarcity of skin at birth. ACC can occur anywhere in the body but majority of cases occur on the scalp. In most cases ACC is an isolated skin defect but some cases might be seen with congenital malformations involving the cardiovascular, gastrointestinal and central nervous systems. ACC is a rare condition with an incidence of 1/10,000 to 3/10,000 births and the exact mechanism is still unknown. Several factors like genetics, teratogens, intrauterin infections can lead to this condition. Most cases occur sporadically but rare familial cases have been reported. Physicians should remember the possible co-occurrence of other congenital anomalies in these infants.

**Figure 1:**
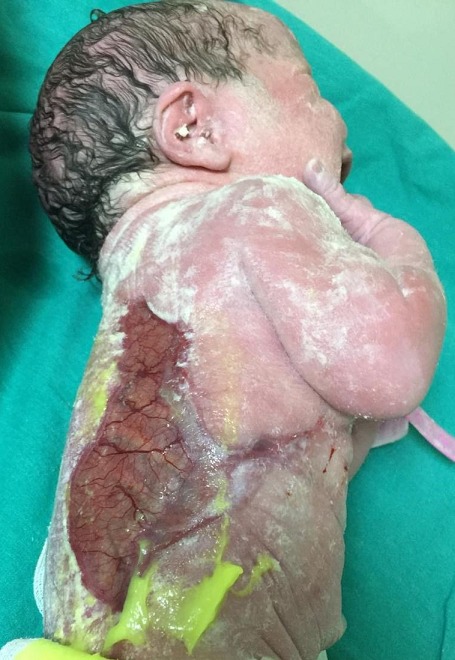
Unilateral absence of skin on the trunk: aplasia cutis congenita

